# Increased ethylene production by overexpressing phosphoenolpyruvate carboxylase in the cyanobacterium *Synechocystis* PCC 6803

**DOI:** 10.1186/s13068-020-1653-y

**Published:** 2020-01-28

**Authors:** Claudia Durall, Pia Lindberg, Jianping Yu, Peter Lindblad

**Affiliations:** 1 Microbial Chemistry, Department of Chemistry-Ångström, Uppsala University, P.O. Box 523, 751 20 Uppsala, Sweden; 2 Biosciences Center, National Renewable Energy Laboratory, Golden, CO USA

**Keywords:** Cyanobacteria, Ethylene, Phosphoenolpyruvate carboxylase (PEPc), Phosphoenolpyruvate synthase (PPSA), Acetyl-CoA

## Abstract

**Background:**

Cyanobacteria can be metabolically engineered to convert CO_2_ to fuels and chemicals such as ethylene. A major challenge in such efforts is to optimize carbon fixation and partition towards target molecules.

**Results:**

The *efe* gene encoding an ethylene-forming enzyme was introduced into a strain of the cyanobacterium *Synechocystis* PCC 6803 with increased phosphoenolpyruvate carboxylase (PEPc) levels. The resulting engineered strain (CD-P) showed significantly increased ethylene production (10.5 ± 3.1 µg mL^−1^ OD^−1^ day^−1^) compared to the control strain (6.4 ± 1.4 µg mL^−1^ OD^−1^ day^−1^). Interestingly, extra copies of the native *pepc* or the heterologous expression of PEPc from the cyanobacterium *Synechococcus* PCC 7002 (*Synechococcus*) in the CD-P, increased ethylene production (19.2 ± 1.3 and 18.3 ± 3.3 µg mL^−1^ OD^−1^ day^−1^, respectively) when the cells were treated with the acetyl-CoA carboxylase inhibitor, cycloxydim. A heterologous expression of phosphoenolpyruvate synthase (PPSA) from *Synechococcus* in the CD-P also increased ethylene production (16.77 ± 4.48 µg mL^−1^ OD^−1^ day^−1^) showing differences in the regulation of the native and the PPSA from *Synechococcus* in *Synechocystis*.

**Conclusions:**

This work demonstrates that genetic rewiring of cyanobacterial central carbon metabolism can enhance carbon supply to the TCA cycle and thereby further increase ethylene production.

## Background

Cyanobacteria are the oldest organisms performing oxygenic photosynthesis. They appeared more than 2.5 billion years ago and were responsible for raising the oxygen levels in the atmosphere from being anoxic to about 20% [1–3]. Cyanobacteria fix inorganic carbon mainly through the Calvin–Benson–Bassham cycle using the inefficient ribulose 1,5-bisphosphate carboxylase/oxygenase (RuBisCO), a similar system as C3 plants [4, 5].

**Fig. 1:**
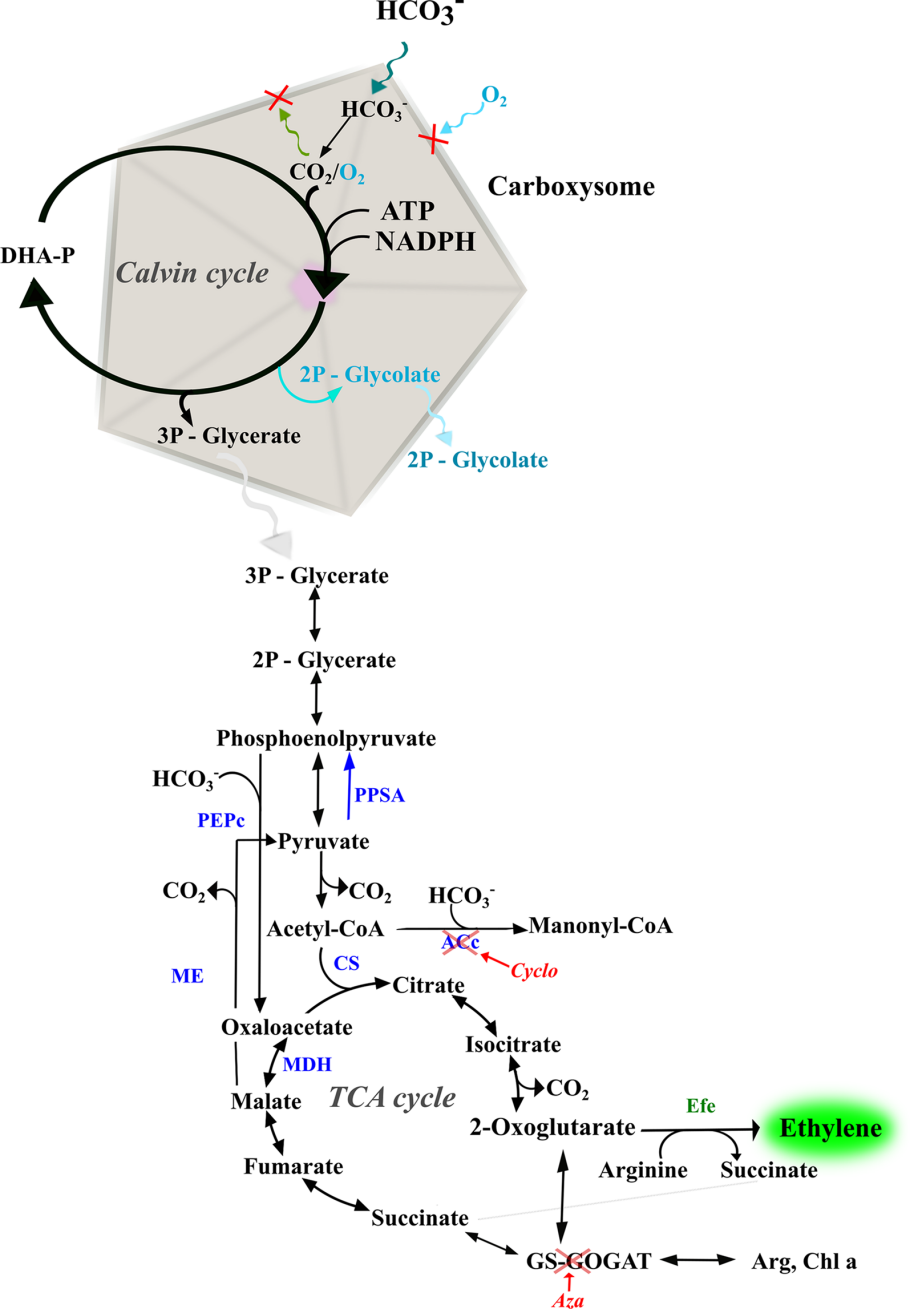
Main carbon fixation metabolism in cyanobacteria. The blue and the green colours correspond to native and non-native enzymes in *Synechocystis* PCC 6803, respectively. ACc: acetyl-CoA carboxylase, Arg: arginine, Aza: azaserine, Calvin cycle: Calvin–Benson–Bassham cycle, Chl a: chlorophyll *a*, CS: citrate synthase, Cyclo: cycloxydim, DHA-P: dihydroxyacetone phosphate, Efe: ethylene-forming enzyme, GS-GOGAT: glutamine synthase glutamine oxoglutarate aminotransferase, MDH: malate dehydrogenase, ME: malic enzyme, PEPc: phosphoenolpyruvate carboxylase, PPSA: phosphoenolpyruvate synthase, TCA cycle: tricarboxylic acid cycle

RuBisCO is the most abundant enzyme on earth and it can perform two competing reactions depending on the substrate used, either CO_2_ or O_2_ [6]. When RuBisCO performs the oxygenase reaction, where O_2_ is produced as a secondary product of the photosynthesis reactions, the metabolite produced (glyoxylate) is toxic for the cells, leading to losing both carbon and energy. This process is called photorespiration [7]. In order to overcome this reaction cyanobacteria have developed a carbon concentrating mechanism (CCM). The CCM consists in five different inorganic transporters (three for HCO_3_^−^ and two for CO_2_), the carboxysome and the carbonic anhydrase. Cyanobacteria have survived in water environments due to the CCM, overcoming low solubility of CO_2_ in water. In water, CO_2_ exists in equilibrium with bicarbonate and carbonate depending on pH. Bicarbonate can diffuse into the carboxysome (a micro-compartment where RuBisCO is encapsulated together with the carbonic anhydrase) while O_2_ is blocked from entering the carboxysome. Inside the carboxysome, carbonic anhydrase converts bicarbonate into CO_2_ which can be used as substrate by RuBisCO. When the product 3-phosphoglycerate is formed, it can diffuse out of the carboxysome and be further metabolized in the cytoplasm [8] (Fig. 1).

Cyanobacteria have the potential to mitigate the rising CO_2_ concentration in the atmosphere since they can function as green cell factories that recycle CO_2_. They are prokaryotic cells and relatively easy to genetically engineer, and as photoautotroph they are easy to grow with no competition of land between food production and biotechnological applications. Thus, these photosynthetic organisms are suitable for a sustainable production of substances of human interest [9, 10]. Cyanobacteria have successfully been genetically engineered to produce different substances such as; fatty acids, omega 3-fatty acids, fatty alcohols, mannitol, lactic acid, hexoses, extracellular polysaccharides, ethanol, propanol, 1-butanol, isobutyraldehyde, isobutanol, isoprene, succinate, ethylene and others [11–14].

It has been shown that by engineering cyanobacteria for increased carbon fixation the cells grow faster with increased carbon fixation and biomass production [15, 16]. This can be combined with cells engineered to produce a selected compound. For instance, *Synechococcus elongatus* PCC 7942 (*S. elongatus*) was successfully genetically engineered to produce isobutyraldehyde and the overexpression of RuBisCO in the engineered strain enhanced the isobutyraldehyde production [13]. The overexpression of selected Calvin cycle genes increased ethanol production in the cyanobacterium *Synechocystis* PCC 6803 (*Synechocystis*) [15]. Furthermore, in a strain of *S. elongatus* engineered to produce 2,3-butanediol, additionally overexpressing enzymes in the pathway between the RuBisCO reaction and pyruvate (PYR) formation led to increased carbon fixation and biofuel production [17].

Given the inefficiency of RuBisCO, alternative carbon fixation pathways have been proposed. In 2014, a synthetic pathway based on the 3-hydroxypropionate bicycle was introduced into *S. elongatus* resulting in a bypass of the photorespiration [18]. In 2010, Bar-Even et al. [19] presented the malonyl-CoA–oxaloacetate–glyoxylate (MOG) pathways, which theoretically are more efficient in fixing carbon than any existing native ones. Interestingly, the enzyme used in these pathways is phosphoenolpyruvate carboxylase (PEPc). PEPc is more efficient to fix carbon dioxide than RuBisCO and it is the enzyme used in C4 and CAM plants. The two most efficient MOG pathways identified were the C4-glyoxylate cycle/alanine option and the C4-glyoxylate cycle/lactate option. These two pathways are identical from step 1 to 6 and differ only in the last steps. As a first step towards implementing these pathways in *Synechocystis*, we overexpressed the native *pepc* and the three first enzymes (phosphoenolpyruvate synthase (PPSA), PEPc and malate dehydrogenase), which are all native in *Synechocystis*. Interestingly, the engineered strain overexpressing *pepc* showed a higher PEPc level, increased chlorophyll *a* content and increased in vitro PEPc activity [20].

Ethylene is a precursor of polyethylene, polystyrene, PVC and even polyester, and its industrial production process (steam cracking) releases significant levels of CO_2_ [21]. Ethylene is also produced by plants and is an important signal molecule involved in germination, fruit ripening and senescence. There are three discovered pathways which synthesize ethylene in nature [22, 23]. In one of these, the *Pseudomonas syringae* ethylene-forming enzyme (Efe) requires only two substrates, 2-oxoglutarate and arginine, resulting in ethylene and succinate as products [21, 23, 24]. 2-Oxoglutarate is an intermediate of the tricarboxylic acid cycle (TCA cycle) and it is the signal molecule for the carbon status in the nitrogen metabolism [25]. *efe* has been heterologously expressed in cyanobacteria and it was believed to be unstable [26–28] until recent studies have demonstrated that the observed instability may be associated with the expression strategies rather than toxicity [29]. In addition, *efe* has been expressed in self-replicative vectors or integrated in the chromosome in different organisms, in *Synechocystis* and *S. elongatus* [21, 26, 30, 31] using different promoters [30–32], RBS [30, 33, 34] and increasing the number of copies of *efe* [33], all resulting in ethylene production. Ethylene production in engineered cyanobacteria is supported by drastic changes in carbon metabolism, including increased flux through PEPc [33]. Thus, increasing the capacity of this key enzyme and other relevant enzymes such as PPSA may lead to increased ethylene productivity.

The aim of this study was to test the hypothesis that genetic rewiring of central carbon metabolism can enhance carbon supply to TCA cycle and ethylene production by introducing *efe* into a *Synechocystis* strain overexpressing PEPc. The resulting ethylene-producing strain was further engineered to overexpress the native PPSA or PPSA from *Synechococcus* PCC 7002 (*Synechococcus*). In addition, extra copies of PEPc from *Synechocystis* and *Synechococcus* were also introduced. The engineered strains were cultivated with different treatments in order to address limiting factors for ethylene production.

## Results

### Expression of efe and engineered strains

The *efe* from *Pseudomonas syringae* expressed in our strains was stable and expressed when the promoter (*nrsB*) was induced with Ni^2+^. The *efe* was introduced into two previously engineered strains containing one copy of the native *pepc* (WT+Km^r^ [20]) and three copies of the native *pepc* (WT+2xPEPc [20]), creating CD-C and CD-P strains, respectively. The CD-P strain was further engineered with another copy of the native *pepc* (4× *pepc*, CD-P1), one extra copy of the native *ppsa* (3× *pepc*, 2× *ppsa*, CD-P2) and an extra copy of the native *pepc* and *ppsa* (4× *pepc*, 2× *ppsa*, CD-P3). The same strategy was used but with the heterologous expression of PEPc and PPSA from the cyanobacterium *Synechococcus* PCC 7002, creating CD-P4 (3× native *pepc*, 1× *pepc* PCC 7002), CD-P5 (3× native *pepc*, 1× *ppsa* PCC 7002) and CD-P6 (3× native *pepc*, 1× *pepc* PCC 7002, 1× *ppsa* PCC 7002). The engineered strains containing *efe* and additional modifications are summarized in Table 2.

### Ethylene production

**Fig. 2:**
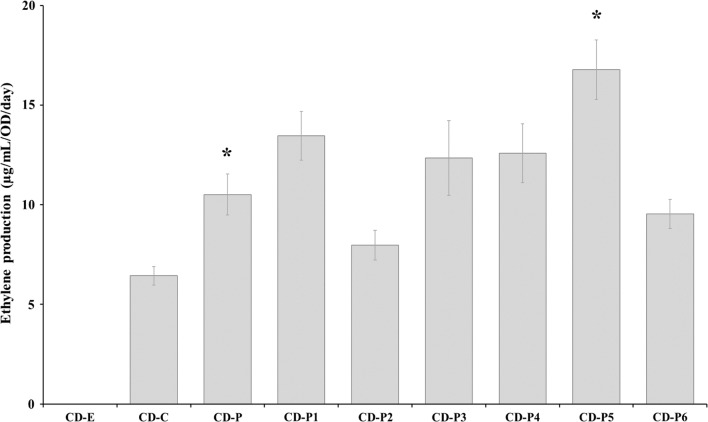
Ethylene production by the *Synechocystis* strains created in this study (Table 2) cultivated under the standard treatment (BG11+50 mM Tris pH 8.0, 50 mM NaHCO_3_, Km (25 µg mL^−1^) Cm (20 µg mL^−1^), 5 µM of NiCl_2_ and 20 µE m^−2^ s^−1^). Asterisks correspond to statistical significant differences compared to CD-C and CD-P, respectively. The experiment was repeated three times with three biological replicates. Error bars represent the mean ± SE

Under the standard treatment (BG11+ 50 mM Tris pH 8.0, 50 mM NaHCO_3_, Km (25 µg mL^−1^) and Cm (20 µg mL^−1^), 5 µM of NiCl_2_ and 20 µE m^−2^ s^−1^, WT and the control engineered strain without the *efe* (CD-E) did not produce any ethylene regardless of Ni^2+^ induction (Fig. 2). The engineered strains containing *efe* did not show ethylene production when the cells were not induced (data not shown). However, when the engineered strains were induced with NiCl_2_ (5 µM) the cells produced ethylene (Fig. 2). Interestingly, significantly higher ethylene productivity was observed in the engineered strain with increased copy numbers of *pepc* (three copies) (CD-P, 10.5 ± 3.1 µg mL^−1^ OD^−1^ day^−1^) compared to cells with WT levels of PEPc (one copy) (CD-C) (6.4 ± 1.4 µg mL^−1^ OD^−1^ day^−1^) (*p* = 0.002) (Fig. 2). When an additional copy of the native *pepc* was introduced in the strain CD-P, leading to the CD-P1 engineered strain, no significant differences of ethylene production was observed (13.5 ± 3.7 µg mL^−1^ OD^−1^ day^−1^). The same result was observed when the native PPSA was overexpressed in the CD-P alone or together with another extra copy of PEPc creating CD-P2 and CD-P3 producing 8.0 ± 2.2 and 12.4 ± 5.7 µg mL^−1^ OD^−1^ day^−1^, respectively. No significant differences in ethylene production were observed between the control strain CD-P and the engineered strains containing the PEPc from the cyanobacterium *Synechococcus*, CD-P4 (12.6 ± 4.4 µg mL^−1^ OD^−1^ day^−1^) and the PEPc and PPSA from *Synechococcus*, CD-P6 (9.5 ± 2.2 µg mL^−1^ OD^−1^ day^−1^). Interestingly, a significant increase in ethylene production was observed when the PPSA from *Synechococcus* was expressed in the CD-P creating the CD-P5 (16.8 ± 4.5 µg mL^−1^ OD^−1^ day^−1^) engineered strain compared to the control strain CD-P (*p* = 0.003) (Fig. 2).

**Fig. 3:**
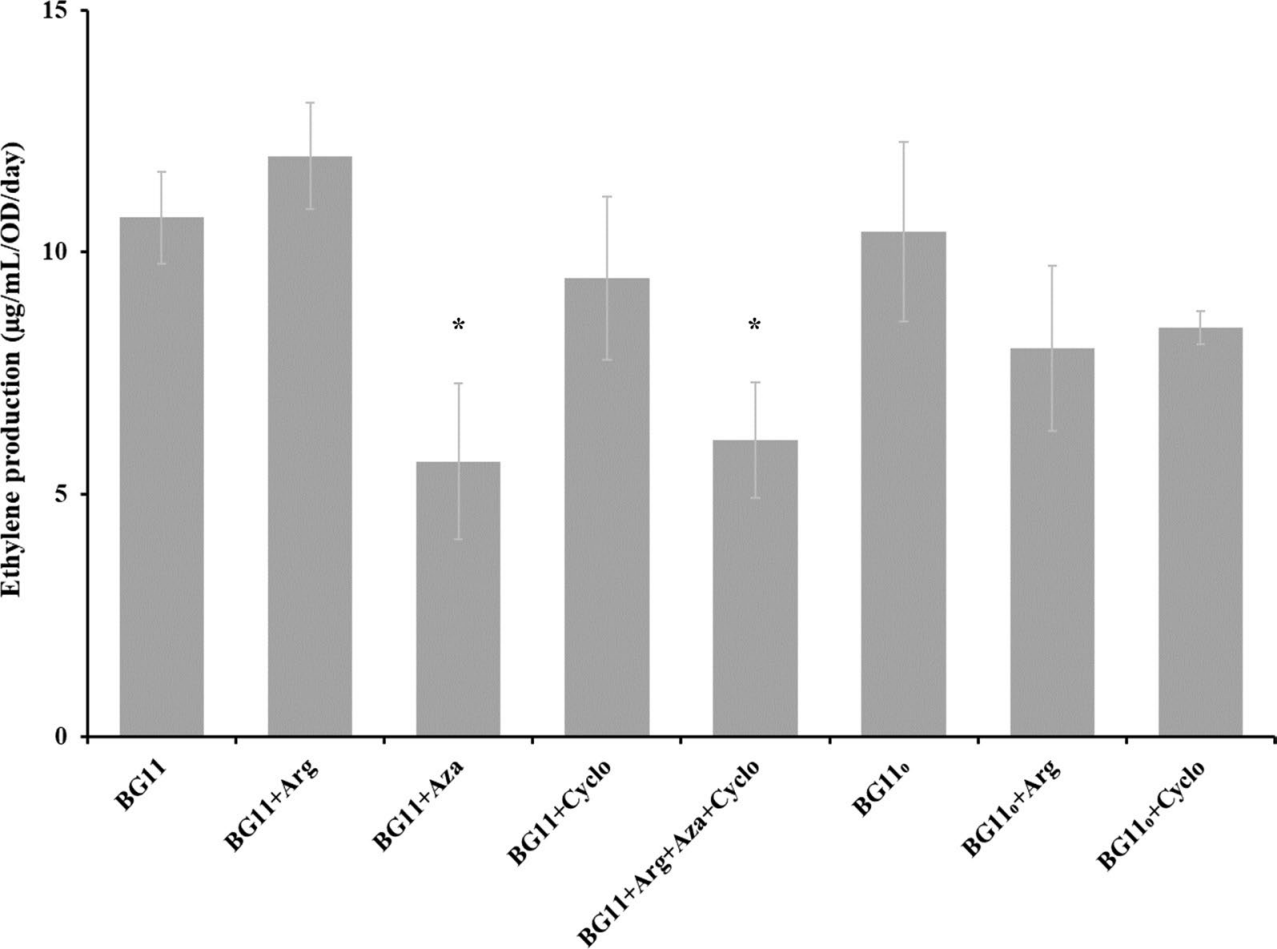
Ethylene production by the *Synechocystis* strain CD-P in different treatments. The different treatments were BG11, BG11_0_, BG11+arginine, BG11_0_+arginine, BG11+azaserine, BG11+cycloxydim, BG11_0_+cycloxydim and BG11+Arg+azaserine+cycloxydim (Table 3) all containing 50 mM Tris pH 8.0, 50 mM NaHCO_3_, Km (25 µg mL^−1^), Cm (20 µg mL^−1^), 5 µM of NiCl_2_ and 20 µE m^−2^ s^−1^. Asterisks correspond to statistically significant differences. The experiment was repeated two times with three biological replicates. Mean ± SE

The ethylene production of the engineered strain, CD-P when different treatments were applied (Table 3) did not show significant differences when BG11+arginine (one of the substrates of Efe) (12.0 ± 3.9 µg mL^−1^ OD^−1^ day^−1^), BG11+cycloxydim (inhibitor of acetyl-CoA carboxylase, [35]) (9.5 ± 4.1 µg mL^−1^ OD^−1^), BG11_0_ (10.4 ± 2.7 µg mL^−1^ OD^−1^ day^−1^), BG11_0_+arginine (8.0 ± 3.4 µg mL^−1^ OD^−1^ day^−1^) and BG11_0_+cycloxydim (8.4 ± 4.2 µg mL^−1^ OD^−1^ day^−1^) compared to the standard treatment, BG11 (10.7 ± 2.3 µg mL^−1^ OD^−1^ day^−1^) (Fig. 3). However, when the cells were treated with BG11+azaserine (an inhibitor of the GS-GOGAT, 35) and BG11+arginine, azaserine and cycloxydim, the ethylene productivity decreased significantly to 5.7 ± 2.9 µg mL^−1^ OD^−1^ day^−1^ (*p* = 0.007) and 6.1 ± 0.8 µg mL^−1^ OD^−1^ day^−1^ (*p* = 0.001), respectively, compared to the standard treatment, BG11 (10.7 ± 2.3 µg mL^−1^ OD^−1^ day^−1^) (Fig. 3).

**Fig. 4:**
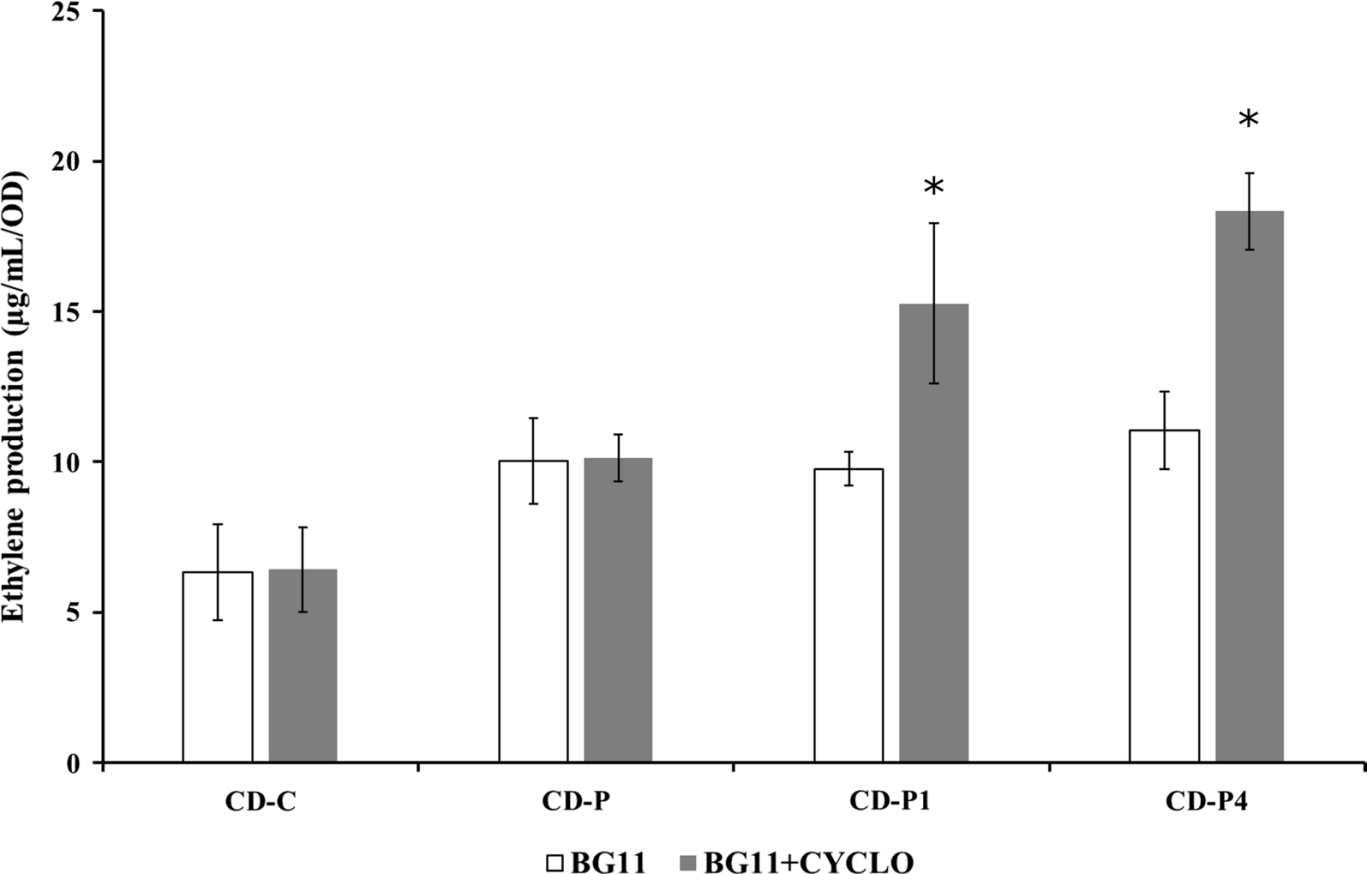
Ethylene production of the *Synechocystis* strains, CD-C, CD-P, CD-P1 and CD-P4 in standard and BG11+cycloxydim treatment (Table 3). Asterisks correspond to statistical significant differences. The experiment was repeated twice with three biological replicates. Mean ± SE

Interestingly, when the engineered strains were incubated with BG11+cycloxydim, no significant differences were observed in ethylene production (data not shown) compared to the standard treatment, BG11, except for the CD-P1 and CD-P4 engineered strains (Fig. 4). CD-P1 and CD-P4 showed significant increased ethylene productivity (19.2 ± 1.3 and 18.3 ± 3.3 µg mL^−1^ OD^−1^ day^−1^, respectively) compared to the standard treatment (*p* = 0.002 and *p* = 0.002, respectively) (Fig. 4).

### Efe and PPSA protein levels

**Fig. 5:**
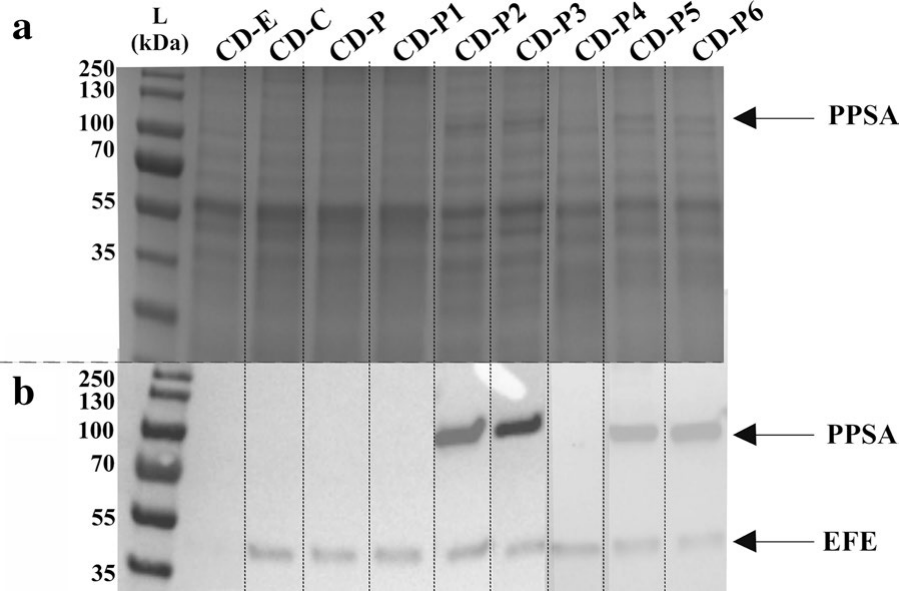
SDS-PAGE/Western immunoblot of the *Synechocystis* strains created in this study (Table 2) analysed for the presence of phosphoenolpyruvate synthase (PPSA) and ethylene-forming enzyme (EFE). *A* SDS-PAGE loaded with 3 µg of protein crude extract from the *Synechocystis* strains created in this study under standard treatment (Table 2) and *B* Western immunoblot using anti-Flag antibody for the *Synechocystis* engineered strains (Table 2) under standard treatment. Standard treatment is BG11+ 50 mM Tris pH 8.0, 50 mM NaHCO_3_, Km (25 µg mL^−1^), Cm (20 µg mL^−1^) and 5 µM of NiCl_2_ and 20 µE m^−2^ s^−1^. The upper band corresponds to PPSA (approximate molecular weight 91.17 and 92.56 kDa for PPSA_6803_ and PPSA_7002_, respectively) and the lower band to EFE (approximate molecular weight 42.16 kDa)

The control strain without *efe* showed no expression of Efe (CD-E) while the Efe protein level was similar in all the induced engineered strains containing *efe* (Table 2) under standard treatment (Fig. 5). The tagged Flag-PPSA was expressed only in the engineered strains (CD-P2, CD-P3, CD-P5, CD-P6) containing PPSA from either *Synechocystis* or *Synechococcus* (Fig. 5).

**Fig. 6:**
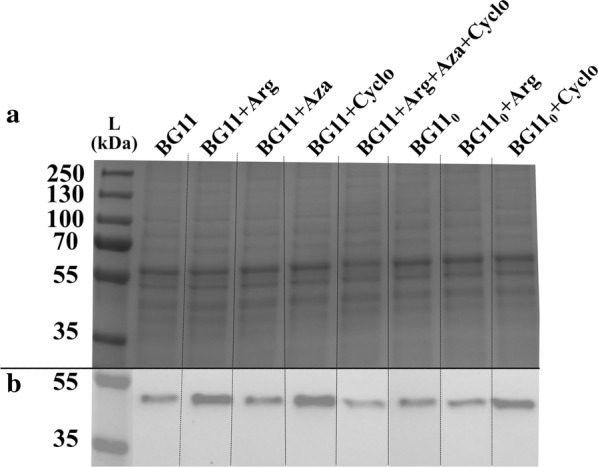
SDS-PAGE/Western immunoblot of the *Synechocystis* strains created in this study (Table 2) analysed for the presence of ethylene-forming enzyme (EFE). A SDS-PAGE loaded with 3 µg of protein crude extract from the *Synechocystis* engineered strain CD-P in different treatments (Table 3) and B Western immunoblot using anti-Flag antibody from the *Synechocystis* engineered strain CD-P in different treatments tested (Table 3). The different treatments were BG11, BG11_0_, BG11+arginine, BG11_0_+arginine, BG11+azaserine, BG11+cycloxydim, BG11_0_+cycloxydim and BG11+arginine+azaserine+cycloxydim (Table 3) all containing 50 mM Tris pH 8.0, 50 mM NaHCO_3_, Km (25 µg mL^−1^), Cm (20 µg mL^−1^), 5 µM of NiCl_2_ and 20 µE m^−2^ s^−1^

When the engineered strain CD-P was incubated with the different treatments, the Efe protein level was similar with the exception of BG11+arginine and the presence of cycloxydim regardless of the presence of nitrate (BG11+cycloxydim and BG11_0_+cycloxydim, Fig. 6). The presence of cycloxydim and arginine in the presence of nitrate increased the Efe protein expression (Fig. 6).

### PEPc protein level

**Fig. 7:**
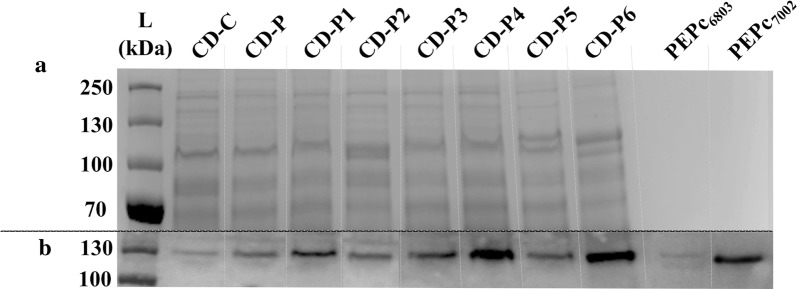
SDS-PAGE/Western immunoblot of the *Synechocystis* strains created in this study (Table 2) analysed for the presence of phosphoenolpyruvate carboxylase (PEPc). *A* SDS-PAGE loaded with 31 µg of protein crude extract from *Synechocystis* strains created in this study under standard treatment (Table 2) and *B* Western immunoblot using anti-PEPc for the *Synechocystis* engineered strains (Table 2) in standard treatment. Standard treatment is BG11+ 50 mM Tris pH 8.0, 50 mM NaHCO_3_, Km (25 µg mL^−1^), Cm (20 µg mL^−1^), 5 µM of NiCl_2_ and 20 µE m^−2^ s^−1^. PEPc_6803_ and PEPc_7002_ corresponds to purified protein (100 ng loaded) of PEPc from *Synechocystis* PCC 6803 and *Synechococcus* PCC 7002, respectively

The PEPc protein level increased with increased number of PEPc copies (Fig. 7). Interestingly, the PEPc protein levels were not affected by the location of the gene in the expression construct, as the levels were similar when the PEPc was expressed alone (CD-P1 and CD-P4) and in the second position in an operon (CD-P3, CD-P6) (Fig. 7). Higher PEPc protein content was observed in the engineered strains expressing PEPc from *Synechococcus* (CD-P4 and CD-P6) than *Synechocystis* (CD-P1 and CD-3) (Fig. 7). However, the affinity of the antibody towards the PEPc from *Synechococcus* was higher than the PEPc from *Synechocystis* (PEPc_6803_ and PEPc_7002_- Fig. 7).

## Discussion

Prior efforts in developing ethylene-producing cyanobacterial strains focused on overcoming genetic instability and increasing Efe expression levels [21, 29, 33, 34]. Here, we show that genetic rewiring of central carbon metabolism can enhance carbon supply to TCA cycle and thereby ethylene productivity.

Other studies have observed that overexpressing PEPc increases the levels of TCA cycle intermediates, for instance, succinate [36, 37]. In 2016, we observed a higher chlorophyll *a* concentration in the cells overexpressing PEPc [20]. Since chlorophyll *a* is synthesized from glutamate, which in turn is formed by 2-oxoglutarate and glutamine, our hypothesis was that this engineered strain had higher 2-oxoglutarate levels and may have the potential of increased ethylene production. The differences in ethylene productivity between CD-C and CD-P were only observed when bicarbonate was present in the media (data not shown). These results suggest that CO_2_ was limiting in the vials, and with bicarbonate addition higher carbon fixation was present in CD-P compared to CD-C resulting in increased ethylene productivity in the CD-P strain.

Interestingly, the overexpression of the native *Synechocystis* PPSA and the heterologous expression of PPSA from *Synechococcus* resulted in differences in ethylene production (CD-P2 and CD-P5, Fig. 2). The DNA and amino acid identity between these proteins are 70.5 and 76.6%, respectively. Little is known about PPSA. It might be that the differences observed in ethylene production are caused by differences in the amino acids sequences in the region located at the N-terminus where ATP is predicted to bind and/or the C-terminus where PYR is predicted to bind, or on the insertion (14 amino acids) present in PPSA from *Synechococcus* (145–160 PPSA PCC 7002 numbering, Additional file 1: Figure S1, Uniprot P23538). According to our own experience, expressing not an exact copy of the native gene (either a codon-optimized version or from another cyanobacterial strain), a heterologous expression of a protein leads to different activities and, not unlikely, regulation than of the native enzyme. Thus, these two proteins may have different kinetics and regulation, leading to different behaviour in the metabolism of *Synechocystis*. In addition, the lower production of ethylene in the CD-P6 strain compared to CD-P5 may be attributed to that when PPSA PCC 7002 is expressed, much of the phosphoenolpyruvat (PEP) component is converted to PYR. However, when PEPc and PPSA PCC 7002 are expressed, less amount of PEP is converted to PYR, then limiting the amount of acetyl-CoA and therefore the carbon flux into the TCA cycle. In order to discover which are the bottlenecks for ethylene production, we decided to investigate the effect of increasing the concentrations of the two substrates of Efe, 2-oxoglutarate and arginine. It has been observed that addition of 2-oxoglutarate did not increase ethylene production in *Synechocystis* but it did in *Synechococcus* [38]. In addition, it is known that when cultivating the cells without a nitrogen source, the 2-oxoglutarate levels increase when a switch in metabolism occurs [21, 39, 40]. A decreased ethylene productivity in an engineered *Synechocystis* strain when the cells were cultivated over several days under nitrogen starvation has been reported [21], however we did not observe this effect in our 24 h incubation (Fig. 3). We also tested the addition of azaserine, which blocks the glutamine amino transferase needed for the GS-GOGAT cycle and therefore increases the 2-oxoglutarate levels [39]. However, less ethylene production was observed in our experiments (Fig. 3), which may be due to arginine metabolism being affected by inhibiting the GS-GOGAT cycle.

In agreement with an earlier study [38], the addition of arginine did not increase the ethylene productivity, thus this amino acid is not a limiting substrate in the engineered strains under our standard treatment. However, when in the same study 2-oxoglutarate and arginine were both added to the cells, ethylene productivity was increased [38]. Thus, the combination of both substrates enhances the ethylene production. However, when we incubated the engineered strain CD-P with BG11_0_+arginine, where the nitrogen levels were very low which should lead to enhanced levels of 2-oxoglutarate, no differences were observed compared to the standard treatment (Fig. 3). It has been reported that nitrogen starvation (and other nutrient stresses) and arginine enhances the production of the nitrogen storage compound cyanophycin [41] and therefore arginine may not have been available for the Efe reaction under this treatment. Ethylene production is also accompanied by accumulation of guanidine inside cyanobacterial cells and in the medium, leaving less nitrogen available for arginine regeneration [42]. More research will be needed to understand the regulation of arginine metabolism in cyanobacteria, and to develop new strategy for improving ethylene production.

Overexpressing PEPc may decrease the amount of PEP. However, it is known that a significant amount of PYR (which can be converted to acetyl-CoA, Fig. 1) is provided by the “C4-like cycle”, which involves PPSA, PEPc, malate dehydrogenase and the malic enzyme [43]. Flux through this pathway is stimulated by ethylene production [33]. Our results further demonstrate that expanding this pathway capacity by enzyme overexpression leads to increased ethylene productivity. Citrate synthase (CS) has a higher affinity for oxaloacetate (the product of PEPc) than acetyl-CoA [44], so if less acetyl-CoA was available, the CS may not catalyse the reaction. Thus, this indicates that acetyl-CoA is either a limiting compound for ethylene production or enhances the PEPc activity (since it is an activator of PEPc [45], when the cells have been engineered with very high levels of PEPc and treated with cycloxydim (CD-P1 and CD-P4) (Fig. 4). Even though the DNA and amino acid sequence showed identities of 66.2 and 63.9%, respectively, and the carboxylases seem to be relatively similar, the in vitro PEPc activity from *Synechococcus* (unpublished) is higher than the activity of PEPc from *Synechocystis* [45], the heterologous activity of the carboxylase may be subject to limitations in substrate availability of *Synechocystis* and therefore no significant differences were observed in ethylene production between CD-P1 and CD-P4.

It was previously described that the Efe protein level was a limiting factor in ethylene production [30, 33]. However, in the engineered strain CD-P, it seems that the level of Efe protein is not limiting ethylene production since the presence of cycloxydim, and possibly increased acetyl-CoA or arginine, upregulated the Efe protein levels without increased ethylene production (Fig. 3). Thus, in this engineered strain and test conditions, other factors limit the production of ethylene.

## Conclusions

In conclusion, genetic rewiring of cyanobacterial central carbon metabolism via overexpression of PEPc and PPSA increased ethylene productivity in *Synechocystis* engineered for the production of ethylene via an introduced Efe. This strategy can be incorporated into strain development for cyanobacterial production of ethylene, as well as of other products derived from the TCA cycle.

## Methods

### Construction of plasmids

To amplify the ethylene gene (*efe*)-Flag tagged, 1–10 ng of plasmid pJU158 [33] (quantified using a Nanodrop 2000 Spectrophotometer, Thermo Scientific) and for amplification of the native genes PEPc and PPSA, 50–250 ng of genomic DNA from *Synechocystis* or *Synechococcus* was used for the Phusion High-Fidelity Hot Spot II DNA polymerase (Finnzymes) as template together with gene-specific primers (Additional file 1: Table S1). The polymerase chain reaction (PCR) products were purified using the Gene JET Purification kit (from Thermo Scientific). Obtained PCR products, the plasmid pEERM3+ (plasmid recombining with the neutral site I of *Synechocystis* and containing the *nrsB* promoter [46, 47] and the plasmid pPMQAC1 (self-replicative plasmid containing the native psbA2 promoter) were digested by selected restriction enzymes (Fermentas) and purified using the DNA clean & concentration™^−5^ from Zymo. Digested PCR product and plasmid were then ligated using the Quick Ligation™ Kit (New England Biolabs) and kept at room temperature for 15 min. The plasmids created are summarized in Table 1.

**Table 1:** Plasmids used/constructed in this study

Plasmid	Promoter	Gene expressed	Resistant cassette	Integration site	References
pEERM	psbA2	–	Km	psbA2	[46]
PEPc-pEERM	psbA2	*pepc* from *Synechocystis* PCC 6803	Km	psbA2	[20]
pEERM3+	nrsB	–	Sp	NSI	[46]
efe-pEERM3+	nrsB	*efe* from *P. syringae*	Sp	NSI	This study
pPMQAC1	psbA2	–	Cm	Self-replicative vector	This study
PEPc_6803_-pPMQAC1	psbA2	*pepc* from *Synechocystis* PCC 6803	Cm	Self-replicative vector	This study
PPSA_6803_-pPMQAC1	psbA2	*ppsa* from *Synechocystis* PCC 6803	Cm	Self-replicative vector	This study
PPSA_6803_+ PEPc_6803_-pPMQAC1	psbA2	*ppsa* and *pepc* from *Synechocystis* PCC 6803	Cm	Self-replicative vector	This study
PEPc_7002_-pPMQAC1	psbA2	*pepc* from *Synechococcus* PCC 7002	Cm	Self-replicative vector	This study
PPSA_7002_-pPMQAC1	psbA2	*ppsa* from *Synechococcus* PCC 7002	Cm	Self-replicative vector	This study
PPSA_7002_+ PEPc_7002_-pPMQAC1	psbA2	*ppsa* and *pepc* from *Synechococcus* PCC 7002	Cm	Self-replicative vector	This study

### Transformation of *Escherichia coli* DH5α

5–6 µL of the ligated DNA product was mixed with 50 µL of thawed ice cold competent cells of *E. coli* DH5α. The tube was incubated on ice for 30 min. After that, the tube was placed at 42 °C for 1 min and incubated on ice again for 5 more min. 450 µL of Luria–Bertani (LB) medium at room temperature was added and mixed. The cells were then incubated at 37 °C for 1 h. Finally, the cells were centrifuged at 13,300 rpm for 2 min and 450 µL of supernatant was discarded. The remaining supernatant was used to resuspend the cells and spread them onto a LB agar plate containing spectinomycin (Sp, 50 µg mL^−1^) or chloramphenicol (Cm, 20 µg mL^−1^) before placed overnight at 37 °C.

Overnight colonies were used to run a PCR in order to verify if they had incorporated the genetic construct. Dream *Taq* DNA polymerase protocol from Fermentas was used together with the primers described above (2.1.). The positive colonies were grown with LB media containing either Sp (50 µg mL^−1^) or Cm (20 µg mL^−1^) and incubated shaking, at 37 °C overnight. Next day, the plasmid was extracted from the cells using the JET Plasmid Miniprep kit from Thermo Scientific. The purified plasmid was sent for sequencing (Eurofins) and when it was confirmed to be correct they were transformed or conjugated to the previously engineered *Synechocystis* strains (WT+Km^r^ and WT+2xPEPc [20] for transformation, and CD-P for conjugation).

### Transformation of *Synechocystis*

*Synechocystis* cells overexpressing the native phosphoenolpyruvate carboxylase (PEPc) (WT+2xPEPc) or containing the kanamycin (Km) antibiotic cassette (WT+Km^r^) were grown in BG11 medium with Km (50 µg mL^−1)^ for 2–3 days. When the cells reached OD_750_ around 0.3 (determined using a Varian 50 Bio UV–Visible Spectrophotometer), 10 mL of cells were then harvested by centrifugation (5000 rpm for 10 min at 20 °C). The pellet was resuspended in 250 µL of BG11. 100 µL of cells were then mixed with 1 µg of plasmid. The mixture was incubated 4–6 h in low light at 30 °C. The cells were spread on a nitrocellulose filter on top of BG11 agar plates. After approximately 24 h, the filters were changed to a BG11 plate containing Km (25 µg mL^−1^) and Sp (10 µg mL^−1^). Positive colonies were confirmed by PCR and re-streaked on plates with increased Sp concentration (up to 50 µg mL^−1^) until PCR did not show any band corresponding to the native neutral site sequence. The cells were grown only with kanamycin for 2 weeks and a PCR confirmed that the ethylene construct was fully segregated. The engineered strains are summarized in Table 2.

**Table 2:** Engineered strains used/constructed in this study

Engineered strains	Plasmid	References
WT+Km^r^	pEERM	[20]
WT+2xPEPc	PEPc-pEERM	[20]
CD-E	pEERM+pEERM3+ +pPMQAC1	This study
CD-C	pEERM + efe-pEERM3+ +pPMQAC1	This study
CD-P	PEPc-pEERM + efe-pEERM3+ +pPMQAC1	This study
CD-P1	PEPc-pEERM + efe-pEERM3+ +PEPc_6803_-pPMQAC1	This study
CD-P2	PEPc-pEERM + efe-pEERM3+ +PPSA_6803_-pPMQAC1	This study
CD-P3	PEPc-pEERM + efe-pEERM3+ +PPSA_6803_+ PEPc_6803_-pPMQAC1	This study
CD-P4	PEPc-pEERM + efe-pEERM3+ +PEPc_7002_-pPMQAC1	This study
CD-P5	PEPc-pEERM + efe-pEERM3+ +PPSA_7002_-pPMQAC1	This study
CD-P6	PEPc-pEERM + efe-pEERM3+ +PPSA_7002_+ PEPc_7002_-pPMQAC1	This study

### Conjugation

1 mL of *E. coli* cells containing the desired plasmids and 1 mL of *E. coli* (pRL443) were grown overnight at 37 °C at 150 rpm with LB medium containing Cm (20 µg mL^−1^) and ampicillin (100 µg mL^−1^), respectively. The cells were mixed in equal ratio and centrifuged for 5 min at 13,000 rpm. The pellet was resuspended in 200 µL of LB and 20 µL of the cells overexpressing PEPc and expressing Efe (CD_P) were mixed and incubated for 2–3 h at 30 °C under low light. The cells were spread on a nitrocellulose filter on top of BG11 agar plates. After approximately 24 h the filters were changed to a BG11 plate containing Km (25 µg mL^−1^), Sp (10 µg mL^−1^) and Cm (20 µg mL^−1^). Positive colonies were confirmed by PCR. The engineered strains are summarized in Table 2.

### Ethylene measurements

50 mL of the engineered *Synechocystis* cells were grown in BG11 with Km (25 µg mL^−1^), Sp (10 µg mL^−1^) and Cm (20 µg mL^−1^) under low light (20 µE m^−2^ s^−1^) until the OD_750_ was between 0.4 and 0.6 (determined using a Varian 50 Bio UV–Visible Spectrophotometer). The cells were harvested at 5000 rpm, for 15 min at room temperature. The pellet was then resuspended in 2 mL of BG11 with Km (25 µg mL^−1^), Cm (20 µg mL^−1^), 50 mM of Tris pH 8.0, 50 mM of NaHCO_3_ and with or without 5 µM of NiCl_2_. Then, the OD_750_ were measured (determined using a Varian 50 Bio UV–Visible Spectrophotometer). The cells were then placed into headspace, 6 mL vial, clear glass-bevelled top and flat bottom vials from Sigma (27,293) and completely sealed. The vials were placed shaking, under 20 µE m^−2^ s^−1^ for 24 h.

After 24 h, 100 µL of the gas phase was collected with a gas syringe and injected into a Clarus 580 Perkin Elmer FID gas chromatograph (GC) with a packed column (1.8 m × 2 mm i.d., Cat No. N9305013-ZW5531, Perkin Elmer). The carrier gas was N_2_ at 20 mL min^−1^. The GC program was 200 °C for 1 min. The area of the ethylene peak at 0.4 min was converted into mg mL^−1^ using the equation obtained from the standard curve. Pure ethylene was injected into the sealed vials and consequent dilutions were made.

When different treatments were used, the pellet was then resuspended in 2 mL of BG11 or BG11_0_ with Km (25 µg mL^−1)^, Cm (20 µg mL^−1^), 50 mM of Tris pH 8.0, 50 mM of NaHCO_3_, 5 µM of NiCl_2_ alone or with 50 µM arginine, 100 µM azaserine or 100 µM cycloxydim or all combined. The treatments used are summarized in Table 3.

**Table 3:** Different treatments used in this study

Treatment	Media	Addition	Action	Effect on the cells
Standard	BG11	–		
BG11_0_	BG11_0_	–	Nitrogen starvation	Higher substrate concentration: 2-oxoglutarate
BG11_0_+Arg	BG11_0_	Arginine	Nitrogen starvation and increased arginine	Higher substrates concentration: 2-oxoglutarate and arginine
BG11+Arg	BG11	Arginine	Increased arginine	Higher substrate concentration: arginine
BG11+Aza	BG11	Azaserine	Inhibition of the glutamine amino transferase in the glutamate synthase (GS-GOGAT cycle)	Higher substrate concentration: 2-oxoglutarate
BG11+Cyclo	BG11	Cycloxydim	Inhibition of acetyl-CoA carboxylase	Higher acetyl-CoA concentration
BG11_0_+Cyclo	BG11_0_	Cycloxydim	Nitrogen starvation and inhibition of acetyl-CoA carboxylase	Higher 2-oxoglutarate and acetyl-CoA concentration
BG11+Arg+ Aza+Cyclo	BG11	Arginine, azaserine and cycloxydim	Increased arginine, inhibition of the glutamine amino transferase in the glutamate synthase (GS-GOGAT cycle) and inhibition of acetyl-CoA carboxylase	Higher arginine, 2-oxoglutarate and acetyl-CoA concentration

In all treatments, 50 mM Tris pH 8.0, 50 mM NaHCO_3_, Km (25 µg mL^−1^), Cm (20 µg mL^−1^) and 5 µM of NiCl_2_ were added and the cells were incubated, 24 h under 20 µmol photons m^−2^ s^−1^

The experiments were done independently at least twice with three biological replicates each. The statistical analysis was performed using the Student’s two-tailed *t* test, *p* < 0.05.

### SDS-PAGE and Western immunoblot (WB)

After the ethylene measurements, 1 or 2 mL of cells were centrifuged (5000 rpm for 10 min at 4 °C) and the pellets were suspended with 2 mL of PBS buffer ([48] and Golden 2007), centrifuged (5000 rpm for 10 min) before pellet resuspended in 200 µL of PBS. Then 3 µL of protease inhibitor (Protease Arrest™ [100×], Biosciences) was added. From this step the samples were always kept on ice. A glass beater (6800 rpm for 30 s, three times, keeping the samples on ice for 5 min in between the 30 s intervals) was used in order to break the cells. 100 µL of PBS buffer was added and the tubes were spun down for 30 s, 12,700 rpm at 4 °C. The supernatants were then transfer into clean Eppendorf tubes and centrifuged for 45 min, 12,700 rpm at 4 °C. The supernatant was then used to measure the soluble protein content of the samples by using the RC DC protein (Bio-Rad) kit. Albumin from bovine serum (Sigma) was used for the standard.

3 µg of protein was loaded on two Mini Protean TGX-Stain free gels (any kDa) from Bio-Rad. The gels were run using a Mini-PROTEAN TGX™ system (Bio-Rad) for 35–40 min at 200 V. One gel was stained using Page Blue Protein staining gel from Thermo Scientific while the other was used for the blot using the Trans-Blot turbo transfer pack and system from Bio-Rad. The transfer was run at 25 V for 30 min. After that the gel was stained with Coomassie blue (control) while the membrane was incubated with 5% non-fat dried milk from AppliChem, for 1 h at room temperature under constant shaking. Then the membrane was washed 15 min, three times with T-TBS. The mouse anti-FLAG antibody (monoclonal mouse from Agrisera (1:1000) was added onto the membrane for 1 h at room temperature under constant shaking. Thereafter, the membrane was washed with T-TBS as described above. The membrane was then incubated with an HRP-conjugated rabbit anti-mouse antibody from Agrisera (1:5000) for 1 h at room temperature under constant shaking. Finally the membrane was washed and developed using the Immuno-Star™ HRP- substrate kit (Bio-Rad) and the chemiluminescence reaction detected using a Chemi Doc XRS machine (Bio-Rad).

For the detection of PEPc, the procedure was the same as mentioned above except that 31 µg of protein was loaded, ECL™ Blocking Agent from GE healthcare was used, incubated overnight at 4 °C, the rabbit anti-PEPc antibody (polyclonal mouse from Agrisera (1:300) was incubated overnight at 4 °C and an HRP-conjugated goat anti-rabbit from Agrisera (1:5000) was used.

### Sequence alignment

The DNA and amino acid identity as well as the sequence alignment of PPSA from *Synechocystis* and *Synechococcus* was done using Clustal Omega.

## Supplementary information

**Additional file 1: Table S1.** Primers used to amplify the ethylene forming enzyme (*efe*), *phosphoenolpyruvate synthase* (PPSA) and *phosphoenolpyruvate carboxylase* (PEPc) from *Synechocystis* PCC 6803 (subscript (6803) and *Synechococcus* PCC 7002 (subscript (7002). The forward primer (operon) has a RBS upstream of the starting codon in order to be placed in an operon after the *phosphoenolpyruvate synthase* (PPSA). PPSA forward primer contains a Flag tag (underlined). **Figure S1.** Sequence alignment of Phosphoenolpyruvate synthase from *Synechocystis* (PPSA PCC 6803) and *Synechococcus* (PPSA PCC 7002). An * indicates positions which have a single, fully conserved residue, : indicates conservation between groups of strongly similar properties—scoring > 0.5 in the Gonnet PAM 250 matrix,. indicates conservation between groups of weakly similar properties—scoring ≤ 0.5 in the Gonnet PAM 250 matrix.

## Data Availability

The data sets used and/or analysed during the current study are available from the corresponding author on reasonable request.

## Abbreviations

Arg: arginine
Aza: azaserine
Cyclo: cycloxydim
EFE: ethylene-forming enzyme
PEPc: phosphoenolpyruvate carboxylase
PPSA: phosphoenolpyruvate synthase
