# Supplementary material for: Increased ethylene production by overexpressing phosphoenolpyruvate carboxylase in the cyanobacterium Synechocystis PCC 6803
Source: Biotechnol Biofuels. 2020 Jan 28;13:16. doi: 10.1186/s13068-020-1653-y (PMC6988332; doi:10.1186/s13068-020-1653-y)
Supplement: Supplementary file 1 — Additional file 1: Table S1. Primers used to amplify the ethylene forming enzyme (efe), phosphoenolpyruvate synthase (PPSA) and phosphoenolpyruvate carboxylase (PEPc) from Synechocystis PCC 6803 (subscript (6803) and Synechococcus PCC 7002 (subscript (7002). The forward primer (operon) has a RBS upstream of the starting codon in order to be placed in an operon after the phosphoenolpyruvate synthase (PPSA). PPSA forward primer contains a Flag tag (underlined). Figure S1. Sequence alignment of Phosphoenolpyruvate synthase from Synechocystis (PPSA PCC 6803) and Synechococcus (PPSA PCC 7002). An * indicates positions which have a single, fully conserved residue, : indicates conservation between groups of strongly similar properties—scoring > 0.5 in the Gonnet PAM 250 matrix,. indicates conservation between groups of weakly similar properties—scoring ≤ 0.5 in the Gonnet PAM 250 matrix. [file 13068_2020_1653_MOESM1_ESM.pdf]

Additional file

Increased ethylene production by overexpressing phosphoenolpyruvate carboxylase in the cyanobacterium *Synechocystis* PCC 6803

Claudia Durall[a], Pia Lindberg[a], Jianping Yu[b] and Peter Lindblad[a]

[a] Microbial chemistry, Department of Chemistry - Ångström, Uppsala University, P.O. Box 523, SE-751 20, Uppsala, Sweden

[b] Biosciences Center, National Renewable Energy Laboratory, Golden, CO, United States

Additional file

Supplementary material

Table S1. Primers used to amplify the ethylene forming enzyme (*efe*), *phosphoenolpyruvate synthase* (PPSA) and *phosphoenolpyruvate carboxylase* (PEPc) from *Synechocystis* PCC 6803 (subscript (6803) and *Synechococcus* PCC 7002 (subscript (7002). The forward primer (operon) has a RBS upstream of the starting codon in order to be placed in an operon after the *phosphoenolpyruvate synthase* (PPSA). PPSA forward primer contains a Flag tag (underlined).

Gene	Forward primer	Reverse primer
Efe	CTCATCTAGAATGACCAATTTGCAAACTTTTG	CAATGGATCCCTTATTTATCATCATCATCTTTGTAATC
PEPc	GTCTTCTAGAATGAACTTGGCAGTTCCTG	CGTACTGCAGGGATCCACTAGTTCAACCAGTATTACGCATTC
PPSA	GTCTTCTAGAATGTCAGGCTCTGACTACAAGGATGACGATGACAAGGTAAGTTCAGTCGTCGAAAAAA	CGTACTGCAGGGATCCACTAGTCTAGCCTAGGGCTTTTTCC
PEPc-operon	GTCTTCTAGATAGTGGAGGTTAGAGAATGAACTTGGCAGTTCCTG	CGTACTGCAGGGATCCACTAGTTCAACCAGTATTACGCATTC
PEPc$_{7002}$	GTCTTCTAGAATGAACCAAGTCATGCATCCC	CGTACTGCAGGGATCCACTAGTTCAACCCGTGTTCCGCAT
PPSA$_{7002}$	GTCTTCTAGAATGTCAGGCTCTGACTACAAGGATGACGATGACAAGGTTAGCACTCTTAACACGGC	CGTACTGCAGGGATCCACTAGTCTATCGACCGAGTTTTTGTT
PEPc$_{7002}$-operon	GTCTTCTAGATAGTGGAGGTTAGAGAATGAACCAAGTCATGCATCCC	CGTACTGCAGGGATCCACTAGTTCAACCCGTGTTCCGCAT

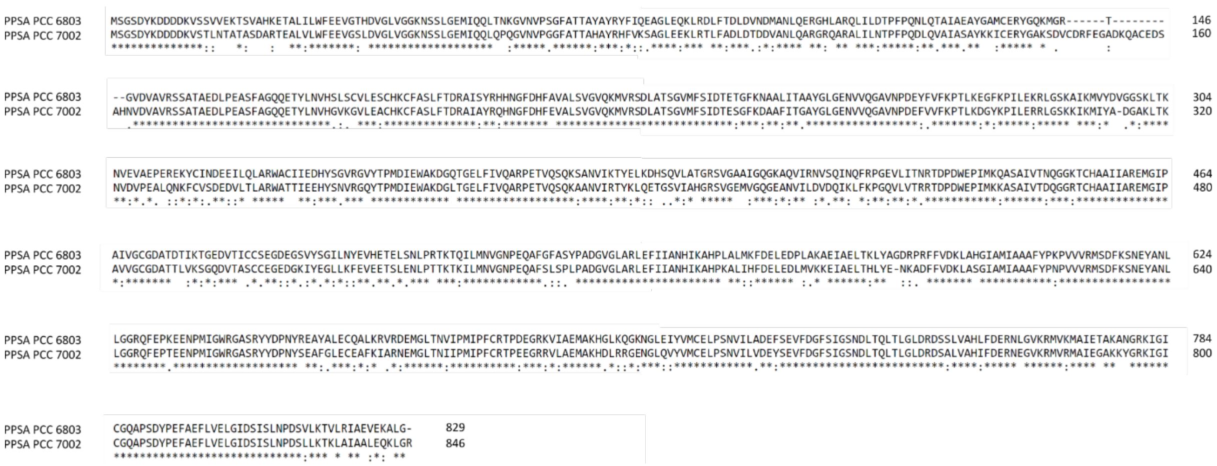

Figure S1. Sequence alignment of Phosphoenolpyruvate synthase from *Synechocystis* (PPSA PCC 6803) and *Synechococcus* (PPSA PCC 7002). An ***** indicates positions which have a single, fully conserved residue, **:** indicates conservation between groups of strongly similar properties - scoring > 0.5 in the Gonnet PAM 250 matrix, **.** indicates conservation between groups of weakly similar properties - scoring =< 0.5 in the Gonnet PAM 250 matrix.
